# Latest Findings of the Regenerative Materials Application in Periodontal and Peri-Implant Surgery: A Scoping Review

**DOI:** 10.3390/bioengineering9100594

**Published:** 2022-10-21

**Authors:** Simone Gallo, Maurizio Pascadopoli, Matteo Pellegrini, Federica Pulicari, Mattia Manfredini, Paolo Zampetti, Francesco Spadari, Carlo Maiorana, Andrea Scribante

**Affiliations:** 1 Unit of Orthodontics and Pediatric Dentistry, Section of Dentistry, Department of Clinical, Surgical, Diagnostic and Pediatric Sciences, University of Pavia, 27100 Pavia, Italy; 2 Section of Dentistry, Department of Clinical, Surgical, Diagnostic and Pediatric Sciences, University of Pavia, 27100 Pavia, Italy; 3 Maxillo-Facial and Odontostomatology Unit, Fondazione IRCCS Cà Granda Ospedale Maggiore Policlinico, 20122 Milan, Italy; 4 Department of Biomedical, Surgical and Dental Sciences, University of Milan, Via della Commenda 10, 20122 Milan, Italy; 5 Implant Center for Edentulism and Jawbone Atrophies, Maxillofacial Surgery and Odontostomatology Unit, Fondazione IRCCS Cà Granda Ospedale Maggiore Policlinico, 20122 Milan, Italy

**Keywords:** alveolar ridge augmentations, furcation defects, peri-implantitis, periodontal resorption, regenerative medicine, tissue engineering

## Abstract

Regenerative dentistry represents a therapeutic modern approach involving biomaterials and biologics such as mesenchymal stem cells. The role of regenerative dentistry is promising in all branches of dentistry, especially in periodontology and implantology for the treatment of bony defects around teeth and implants, respectively. Due to the number of different materials that can be used for this purpose, the aim of the present review is to evidence the regenerative properties of different materials both in periodontitis and peri-implantitis as well as to compare their efficacy. Clinical trials, case-control studies, cross-sectional studies, and cohort studies have been considered in this review. The outcome assessed is represented by the regenerative properties of bone grafts, barrier membranes, and biological materials in the treatment of intrabony and furcation defects, peri-implantitis sites, alveolar ridge preservation, and implant site development. Based on the studies included, it can be stated that in the last years regenerative materials in periodontal and peri-implant defects treatments have shown excellent results, thus providing valuable support to surgical therapy. To achieve optimal and predictable results, clinicians should always consider factors like occlusal load control, prevention of microbial contamination, and wound dehiscence. Further evidence is required about the use of enamel matrix derivative in alveolar ridge preservation, as well as of stem cells and bone morphogenetic proteins-2 in furcation defects and peri-implantitis sites. Considering the high amount of research being conducted in this field, further evidence is expected to be obtained soon.

## 1. Introduction

Bone formation is a crucial step in the regeneration of alveolar bone around teeth and dental implants. This process is mainly regulated by different cells and molecular mechanisms encompassing matrix interactions. In recent years, regenerative medicine has shown a wide development with more and more in vitro and clinical studies conducted on this topic [1]. Tissue engineering has also been applied to dentistry, especially in the field of periodontology and implantology. Tooth loss as well as hard and soft tissue remodeling are the direct consequences of an inflammatory process [2]. Bone defects in the mouth can significantly vary as regards their size, starting from small intrabody defects related to periodontal/peri-implant disease until more extended lesions, resulting from trauma and neoplastic lesions. The goal of periodontal and peri-implant tissue engineering is to promote the regeneration of the supporting tissues around teeth or implants. Scaffolds, growth factors, and stem cells exert a key role in the regenerative process, with a synergic effect between them [1].

The first approach that has been used for periodontal regeneration is represented by guided tissue regeneration (GTR), which is based on the use of barriers selecting specific cells to promote their colonization of the periodontium after surgery [3]. Moreover, the use of barriers can be combined with the use of bone substitutes, preventing epithelial migration [4]. Accordingly, the colonization of this protected site by periodontal ligament cells should be the expected outcome.

Following this former technology, decades of research have led to the development of several different biomaterials for alveolar bone regeneration which mainly differ in their mechanism of action. Barriers of different types, bone fillers, and biologics are examples of strategies that clinicians can benefit from to obtain periodontal and peri-implant regeneration [1]. As previously stated, barriers cover periodontal defects, protecting them from epithelial downgrowth. Bone fillers are scaffolds or bone grafts replacing the missing bone inside an alveolar defect. Finally, biologics encompass growth factors, mesenchymal cells, or further substances that can be directly administered in the defect, and thus, promote bone regeneration [5,6].

Considering the wide research conducted in the last years in this evolving field, the aim of the present work is to review the evidence on the regenerative properties of different materials both in periodontitis and peri-implantitis as well as to compare their efficacy.

## 2. Materials and Methods

### 2.1. Focused Questions

What are the regenerative properties of bone grafts, barrier membranes, and biological materials in the treatment of periodontal defects? If any, are these properties applicable to peri-implant defects, alveolar ridge preservation, and implant site development? Are the regenerative properties of these materials similar to each other?

### 2.2. Eligibility Criteria

The inclusion criteria applied in this review were: (I) study design—clinical trials, case-control studies, cross-sectional studies, and cohort studies, (II) participants—periodontal patients with intrabony and furcation defects, patients with peri-implantitis, patients with extraction socket, and patients with crestal bone defects, (III) interventions—use of bone grafts, barrier membranes, and biological materials for the regenerative treatment of intrabony and furcation defects, peri-implantitis sites, alveolar ridge preservation, and implant site development, and (IV) outcome—regenerative properties of bone grafts, barrier membranes, and biological materials in the treatment of intrabony and furcation defects, peri-implantitis sites, alveolar ridge preservation, and implant site development. Studies that did not meet all the inclusion criteria were not considered. Moreover, the exclusion criteria were: (I) abstract of articles published in non-English languages, (II) duplicate studies, (III) not pertinent studies, (IV) in vitro or animal clinical studies, (V) absence of Ethics Committee approval, and (VI) narrative reviews, scoping reviews, systematic reviews, or systematic and meta-analysis reviews.

### 2.3. Search Strategy

According to the JBI methodology for scoping review, a three-step searching process has been considered consisting of the following phases: (i) preliminary limited search on PubMed (MEDLINE) and Scopus; (ii) selecting key terms from retrieved articles for devising search strategy; (iii) searching reference list of all included articles for additional research [7].

Moreover, the PCC model was applied, which is based on the following three elements: population (people with periodontal/peri-implant disease), concept (regenerative materials in oral surgery), and context (in this case, the review has not been limited to any specific cultural factor or setting). Abstracts of studies that evaluated regenerative properties of bone grafts, barrier membranes, and biological materials in the treatment of intrabony and furcation defects, peri-implantitis sites, alveolar ridge preservation, and implant site development were reviewed.

A literature search was performed in the electronic databases PubMed (MEDLINE) and Scopus, searching the following medical subject heading (MeSH): alveolar ridge augmentations, furcation defects, peri-implantitis, periodontal resorption, regenerative medicine, and tissue engineering. The full electronic search strategy is listed in the Supplementary Materials Section.

In order to review the latest evidence on the topic, only articles published in the years 2000 to 2022 were considered, with a data extraction period of 12 weeks. Three reviewers (M.Pa., M.Pe. and M.M.) performed the search, which was last performed on 4 September 2022. Any contrary opinions were resolved by consulting three other reviewers (F.S., C.M. and A.S.). Relevant articles were reviewed by reading the full texts, recording the results, and identifying any similar studies that met the inclusion criteria. Non-relevant studies were excluded by thoroughly analyzing the titles and abstracts of the articles searched.

### 2.4. Quality Assessment of Included Studies

This review was carried out by assessing the risk of bias by performing quality analysis of the clinical studies through the National Heart, Lung, and Blood Institute (NHLBI) Quality Assessment of Controlled Intervention Studies, for Observational Cohort and Cross-Sectional Studies, for Case Series Studies, and Case Reports Studies [8].

## 3. Results

**Figure 1:**
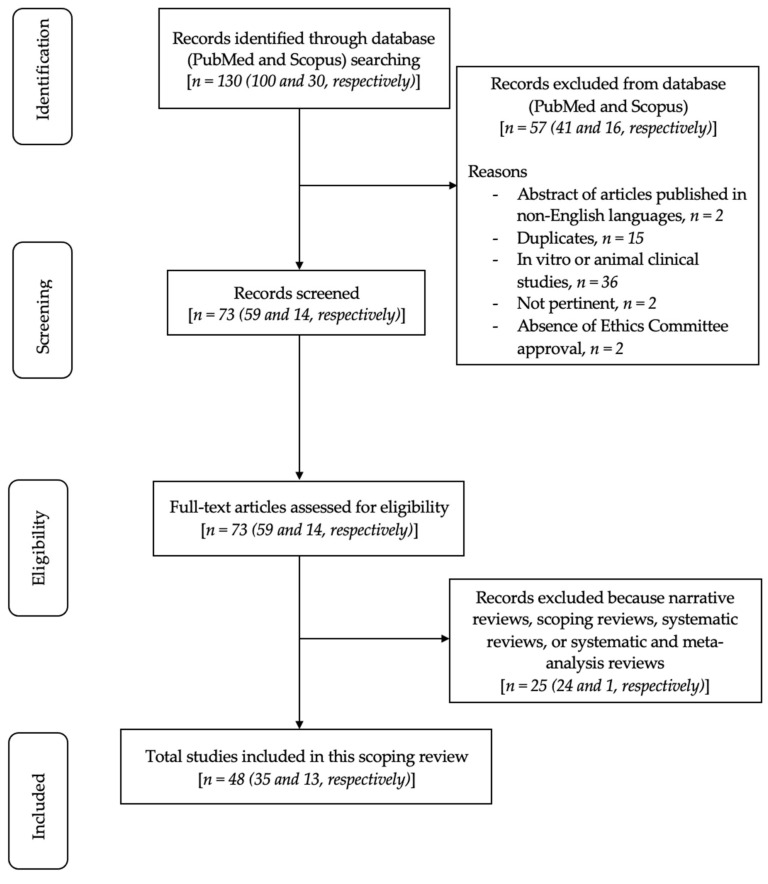
Flowchart of the review process.

In total, 130 articles were primarily identified using MeSH terms. Subsequently, 41 articles from PubMed and 16 articles from Scopus were removed because the abstracts were published in non-English, and/or duplicate, and/or in vitro or animal clinical studies, and/or irrelevant, and/or lacked ethics committee approval. Consequently, 73 articles were screened and assessed for eligibility. Finally, 25 articles were further excluded as narrative reviews, scoping reviews, systematic reviews, or systematic and meta-analysis reviews. In total, 48 relevant articles were included and assessed in this review. The flowchart of the review process is shown in Figure 1.

The studies were from four categories: controlled intervention studies [9,10,11,12,13,14,15,16,17,18,19,20,21,22,23,24,25,26,27,28,29,30,31,32], observational cohort and cross-sectional studies [33,34,35,36,37,38,39,40], and case series/case reports studies [41,42,43,44,45,46,47,48,49,50,51,52,53,54,55,56].

Table S1 (Supplementary Materials) shows the studies excluded from this review and the reasons for exclusion [57,58,59,60,61,62,63,64,65,66,67,68,69,70,71,72,73,74,75,76,77,78,79,80,81].

### Risk of Bias

The Cochrane Collaboration tool was applied to assess the risk of bias of the articles included in this review (Table 1), using the judging criteria for risk of bias shown in Table S2 (Supplementary Materials). A moderate risk of bias is observed in this review.

NHLBI Quality Assessment of Controlled Intervention Studies is shown in Table S3 (Supplementary Materials). NHLBI Quality Assessment Tool for Observational Cohort and Cross-Sectional Studies is shown in Table S4 (Supplementary Materials). NHLBI Quality Assessment Tool for Case Series Studies/Case Reports are shown in Table S5 (Supplementary Materials).

## 4. Discussion

Research performed in vitro and clinical studies regarding regenerative medicine and tissue engineering in dentistry has developed a wide range of performance biomaterials available to the clinician to achieve bone and soft tissue regeneration in the presence of periodontal and peri-implant defects [6].

They can be divided into bone grafts, barrier membranes (autogenous buccal fat pad, allogenic amnion membrane, xenogenic bovine-derived, alloplastic polytetrafluoroethylene, β-tricalcium phosphate, freeze-dried demineralized bone, and titanium) and materials with biological activity, i.e., bioactive factors (EMD, enamel matrix derivative; PRF, platelet-rich fibrin; PRP, platelet-rich plasma; BMP, bone morphogenetic proteins; GF, growth factors), stem cell therapies, and gene therapy through bone morphogenetic proteins [1].

Some materials have been extensively tested while other materials have reported interesting results in recent years.

### 4.1. Periodontal Application: Intrabony Defects

Regenerative periodontal therapy with EMD in association with autogenous bone graft (ABG) or with bovine-derived bone substitutes in the treatment of intrabony defects (IBDs) in patients with chronic periodontitis results in the former case in improved vertical bone resorption, and in the latter case, in significant clinical attachment level (CAL) gain and probing pocket depth (PPD) reduction, with negligible increase in gingival recession (GR) [9,41].

In addition, combined treatment with PRF and ABG in IBDs results in similar CAL gain, PPD reduction, GR increase, and defect bone level gain compared with the therapeutic combination with EMD and ABG [10]. Combination treatment with PRP and demineralized bone matrix has also resulted in significant improvements in IBDs [42]; however, it has recently been shown that the combination of 1.2 mg of Rosuvastatin, a new synthetic, hydrophilic statin with potent anti-inflammatory and osteogenic differentiation actions, ABG, and PRF reduces PPD, CAL, and defect height and diameter more than the application of ABG and PRF alone [11].

Autogenous bone harvesting can also be performed from a mandibular torus, which can ensure adequate filling of IBDs in periodontal patients, avoiding the second surgical site and showing reduced CAL and complete resolution of tooth mobility [43].

It has been observed how the treatment of IBDs, in patients with aggressive periodontitis, by application of xenogeneic grafts plus modified perforated membranes (MPMs) achieves better results when compared with the application of xenogeneic grafts plus standard collagen membranes (CMs): significant reduction in PPD and, in addition, a significant increase in bone density, which could indicate a greater area of new bone formation [12]. Furthermore, periodontal defect coverage with MPMs is associated with a significant initial increase in bone morphogenetic protein-2 (BMP-2) levels in gingival crevicular fluid, which could improve clinical outcomes of tissue-guided regenerative surgery [13]. Finally, PRF has recently been shown to present comparable results to CM for the treatment of IBDs in patients with aggressive generalized periodontitis and better results regarding the position of the gingival margin [14].

Alloplastic bone grafts have also been tested in the treatment of IBDs: in particular, it has been shown that the application of a premixed composite of bioactive calcium phosphosilicate particles and an absorbable synthetic binder in combination with PRF resulted in a greater CAL increase and PPD reduction than the use of premixed composite alone [15].

The addition of autogenous platelet concentrates (APC) to GTR in deep IBDs does not cause a statistically significant improvement in CAL and the long-term stability of results [16].

Furthermore, the application of an allogenic barrier membrane in the treatment of IBDs allows for a significant reduction in periodontal indexes and radiographic measurement of the bone defect area, achieving results such as the use of a demineralized bone matrix allograft [17].

Recently, stem cells in dentistry have also found great use with excellent results. In the treatment of IBDs, autologous stem cells from dental pulp and periodontal ligament, delivered in a collagen scaffold, significantly improved clinical (PPD and CAL) and radiographic parameters of periodontal regeneration [18,19].

Regarding gene therapy in periodontal defects, the literature to date is very poor. However, the only controlled clinical study by Gonçalves et al. [20] showed that root cementum can modulate the expression of mineral-associated growth factors during periodontal regeneration in IBDs.

### 4.2. Periodontal Application: Furcation Defects

It has been shown that in grade II furcation defects, the combination of PRF with β-tricalcium phosphate allograft (β-TCP) results in significant improvements compared to the use of alloplastic graft alone, showing a more significant reduction in horizontal defect depth and vertical defect depth and improved CAL [21]. In addition, the use of PRP also leads to similar results to the application of PRF, with no significant differences [22]. In contrast, EMD and β-TCP association does not provide a significant advantage compared to isolated approaches in the treatment of grade II furcation defects [23].

Furthermore, the application of freeze-dried demineralized bone allograft (DFDBA) in association with the amniotic allogeneic barrier also results in significant improvement of clinical and radiographic parameters in the treatment of grade II furcations compared to the use of DFDBA alone [24]. Similar results have been obtained by applying concentrated GF with an inorganic bovine bone xenograft [25].

Regarding gene therapy, significant clinical and radiographic results regarding the regenerative potential of BMP-2 impregnated with absorbable collagen sponge and PRF in the treatment of grade II furcation defects have been highlighted, and thus, it has been considered as a potential graft material to be used for the treatment of grade II furcation defects [26].

The association of allogenic cancellous bone grafts and PRF can also be applied in the treatment of grade III mandibular furcation, showing PPD reduction, CAL increase, and radiographic bone filling [33,44]. Among autogenous barrier membranes, the pedicled buccal fat pad (PBFP) can be used to increase the predictability and outcome of root coverage procedures in the treatment of III-class GR with furcation involvement [45].

To date, no clinical trial has been conducted aimed to assay the effect of stem cells in furcation defects.

### 4.3. Implant-Based Application: Treatment of Peri-Implantitis

A 50:50 mixture of ABG and allograft can be successfully used in the treatment of circumferential and semicircumferential bone defects in patients with peri-implantitis; in particular, significant improvement in GR, plaque index (PI), PPD, bleeding on probing (BoP), and CAL at 6 and 12 months has been shown [34].

ABG can also be used in combination with an allogenic graft material, titanium mesh, and acellular dermal matrix, leading to significant improvement regarding mean marginal bone loss and bone gain in peri-implant defects [35]. It was shown that the regenerative approach consisting of the application of deproteinized bovine bone mineral and a collagen membrane after mechanical and chemical decontamination with tetracycline solution in the peri-implant defects showed long-term stability of physiologic PPD, with no clinical signs of peri-implant inflammation and BoP and no radiographic bone loss [46]. Furthermore, after mechanical debridement of the implant surface and application of antimicrobial photodynamic therapy, promising results were achieved in terms of absence of granulation tissue, purulence, progressive bone resorption in the presence of newly formed bone tissue in close contact with the implant surface by the application of 70:30 mixture of ABG and deproteinized bovine bone mineral particles, and the use of titanium mesh and resorbable collagen membrane [47].

Finally, complete bone filling of peri-implant bone defect was observed clinically and radiographically by applying a xenogenic bone substitute with dehydrated and deepithelialized human amnion-chorion membrane [48].

Regarding the application of EMD in the treatment of peri-implantitis, the randomized controlled clinical trial by Isehed et al. [27] observed positive clinical and radiographic results in implant survival at 3 years (100%) and 5 years (85%) after surgical treatment. In addition, bovine-derived hydroxyapatite and EMD have also been tested in patients with peri-implantitis, achieving long-term resolution of BoP and suppuration and significant reduction of PPD [49].

To date, no randomized controlled clinical trials have been conducted to test the effect of stem cells and gene therapy in cases of peri-implantitis. Only a case report by Jensen et al. [50] reported improvement in peri-implantitis by applying BMP-2.

### 4.4. Alveolar Ridge Preservation and Implant Site Development

Autogenous mandibular bone blocks covered with a membrane of bone mineral and bovine collagen can be used to regenerate the alveolar process of patients with edentulous and atrophic ridges, with implant survival rates consistent with those obtained for implants placed in native bone [36].

Furthermore, alveolus preservation by application of demineralized bovine bone mineral particles coated with a porcine-derived non-cross-linked collagen matrix was shown to be an effective technique for maintaining long-term stable hard and soft tissue dimensional volumes [37].

To date, the literature suggests that EMD does not provide significant results in alveolar ridge preservation, while highlighting its role in reducing postoperative pain and swelling following dental extraction [28].

Recently, the application of BMP-2-soaked absorbable collagen sponge or BMP-2-dipped tricalcium phosphate and hydroxyapatite particles in alveolar ridge preservation showed significant and similar results [29].

The application of bovine bone combined with a barrier membrane with or without fibroblast growth factor-2 (FGF-2) on dental alveoli can effectively reduce ridge absorption, especially ridge width, while the FGF-2-modified membrane seems to improve the results obtained by using the membrane alone [30]. Similar results in terms of horizontal preservation of the alveolar ridge have been obtained by application of a bovine bone graft covered with anodized titanium foil [51], deproteinized bovine bone mineral with 10% collagen covered with a collagen membrane [38], and xenograft-derived bovine bone mineral and covered with a newly developed dense polytetrafluoroethylene membrane [39].

Particulate ABG and inorganic minerals derived from bovine bone with a 3:7 ratio can be used to perform the sagittal sandwich technique in sinus lift, providing some safety and predictability in the subsequent implant placement [52]. In contrast, the application of allogenic bone blocks grafts to increase pre-implant bone thickness is associated with frequent complications such as graft loss and/or peri-implantitis [53]. Moreover, the cumulative survival rate (CSR) of 192 implants placed in association with guided bone regeneration (GBR) procedures using demineralized bovine bone mineral grafts (DBBM), autologous bone grafts, and a 1:1 ratio mixture of autologous grafts and DBBM (with resorbable or non-resorbable membranes) was compared, achieving satisfactory long-term results with a CSR >90% with all materials used [40].

Recently, it has been shown that the use of composite bone grafts (autogenous bone and xenograft particles) in association with recombinant human platelet-derived growth factor (PDGF) achieves simultaneous vertical ridge augmentation and periodontal regeneration [54]. In addition, both the application of alloplastic poly-lactic-glycolic acid graft coated with calcium β-phosphate (PLGA-β-TCP) and DFDBA particles covered with a rapidly absorbable collagen dressing result in comparable results in terms of maintenance of alveolar bone size, feasibility of implant placement, implant survival, and stability of peri-implant bone level up to 12 months after loading [31].

The application of adipose-derived stem cells derived from buccal fat pad in combination with natural bovine bone mineral can be considered an effective treatment for bone regeneration in large alveolar bone defects, providing implant rehabilitation survival [55].

The prospective case series by Windisch et al. [56] showed efficacy in the survival and predictability of implant rehabilitation following horizontal and vertical GBR using titanium-reinforced high-density polytetrafluoroethylene membranes combined with autogenous bone and particulate bovine-derived xenograft in a 1:1 ratio.

The use of an allograft cellular bone matrix, containing native mesenchymal stem cells and osteoprogenitors, also leads to significant results in bone formation following sinus lift procedures, encouraging faster initiation of implant placement [32].

A summary of the results of studies included in this work is shown in Table 2 Moreover, Table 3 shows a summary of the most relevant growth factors, their origin, composition, mechanism of action, and clinical indications.

This report has some limitations. No information specialists or academic librarians have been included for the conduction of the electronic search. It is possible that the search methodology could have been too specific for a scoping question. Moreover, it did not consider all articles in the literature, with reference to grey literature and non-indexed research. Furthermore, the results may differ according to the population considered. Another limitation could be that the same material used in regenerative dentistry/tissue engineering can be produced by different companies; as this is not always specified in the articles, this aspect should be included, as some differences in the results could occur. Finally, the heterogeneity of commercially available materials, which are often used in combination in the treatment of periodontal and peri-implant defects, complicates the comparison between them.

Future research prospects could include further randomized controlled clinical trials that should be conducted to evaluate the effects of stem cells and gene therapy in the treatment of furcation defects and peri-implantitis. In addition, further studies will be needed to evaluate the possible interactions of regenerative materials with peri-implant probiotic therapy, considering the different populations of microorganisms at peri-implant sites [83] and the supposed competitive role of probiotics against peri-implant pathogens [84]. Though this research has been focused on clinical trials, systematic reviews based on in vitro studies could also be valuable to transfer basic research into clinical application.

## 5. Conclusions

In the last few years, several randomized clinical trials have been carried out to evaluate the outcomes of regenerative materials in periodontal and peri-implant defects treatments, obtaining excellent results, and thus, providing valuable support to surgical therapy. The clinician must always consider occlusal load control, prevention of microbial contamination, and wound dehiscence to achieve optimal and predictable results using such materials.

Further evidence is required about the use of EMD in alveolar ridge preservation, and clinical studies are needed regarding the utility of stem cells and BMP-2 in furcation defects and peri-implantitis sites. However, since the advances made in materials science and tissue engineering in recent years have been considerable, it is fair to expect that in a short time, these issues will also be bridged.

## Figures and Tables

**Table 1:** Risk of bias of the studies included in this review: the green symbol represents a low risk of bias, while the yellow symbol represents a high risk of bias.

	Random Sequence Generation	Allocation Concealment	Blinding	Incomplete Outcome Data	Selective Reporting
Aslan et al., 2020 [9]	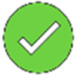	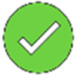	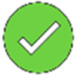	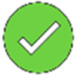	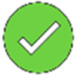
Paolantonio et al., 2020 [10]	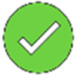	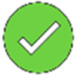	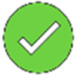	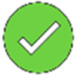	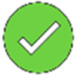
Gautam et al., 2022 [11]	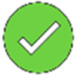	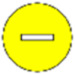	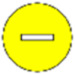	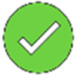	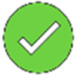
Górski et al., 2020 [12]	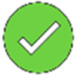	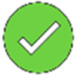	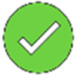	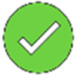	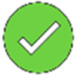
Gamal et al., 2014 [13]	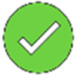	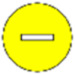	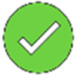	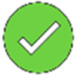	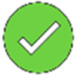
Aggour et al., 2017 [14]	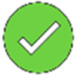	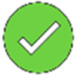	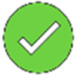	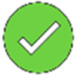	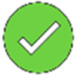
Hazari et al., 2021 [15]	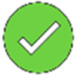	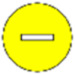	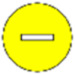	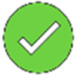	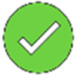
Cieplik et al., 2018 [16]	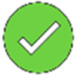	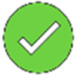	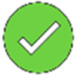	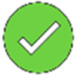	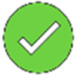
Temraz et al., 2019 [17]	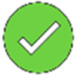	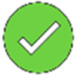	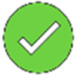	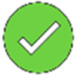	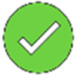
Ferrarotti et al., 2018 [18]	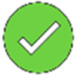	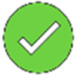	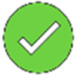	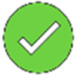	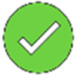
Chen et al., 2016 [19]	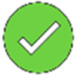	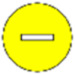	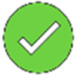	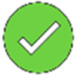	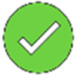
Gonçalves et al., 2008 [20]	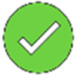	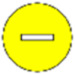	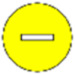	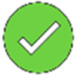	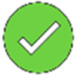
Rani et al., 2018 [21]	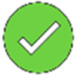	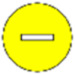	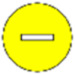	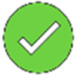	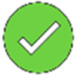
Bajaj et al., 2013 [22]	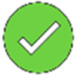	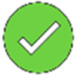	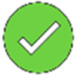	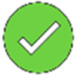	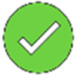
Queiroz et al., 2016 [23]	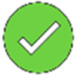	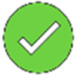	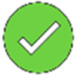	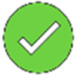	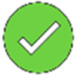
Pajnigara et al., 2017 [24]	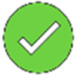	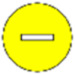	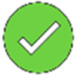	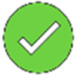	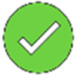
Huidrom et al., 2022 [25]	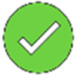	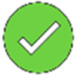	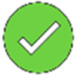	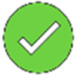	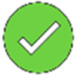
Sneha et al., 2021 [26]	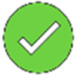	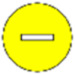	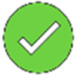	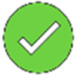	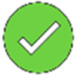
Isehed et al., 2018 [27]	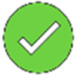	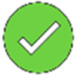	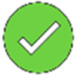	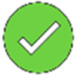	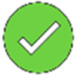
Lee et al., 2020 [28]	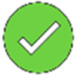	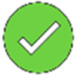	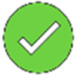	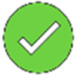	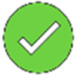
Jo et al., 2019 [29]	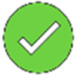	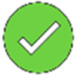	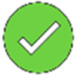	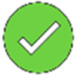	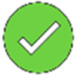
Stumbras et al., 2021 [30]	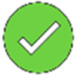	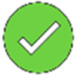	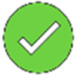	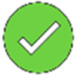	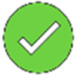
Saito et al., 2021 [31]	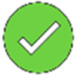	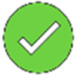	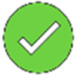	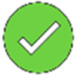	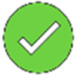
Gonshor et al., 2011 [32]	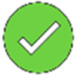	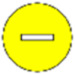	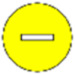	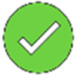	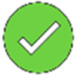
Majzoub et al., 2020 [33]	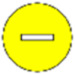	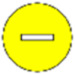	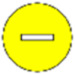	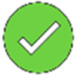	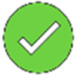
Canullo et al., 2019 [34]	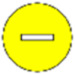	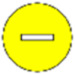	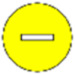	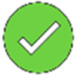	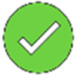
Kadkhodazadeh et al., 2021 [35]	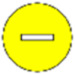	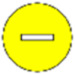	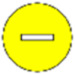	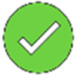	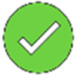
Chiapasco et al., 2020 [36]	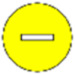	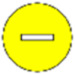	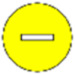	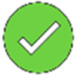	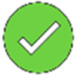
Beretta et al., 2021 [37]	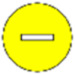	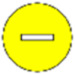	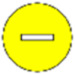	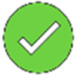	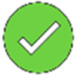
Manavella et al., 2018 [38]	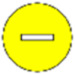	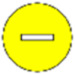	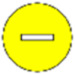	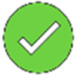	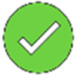
Zafiropoulos et al., 2020 [39]	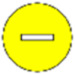	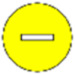	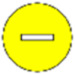	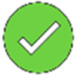	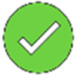
Beretta et al., 2015 [40]	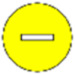	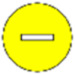	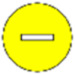	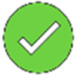	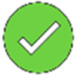
Yoshikawa et al., 2020 [41]	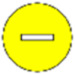	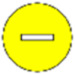	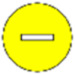	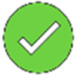	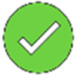
Thakkalapati et al., 2015 [42]	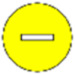	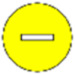	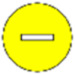	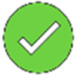	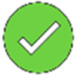
Pal et al., 2018 [43]	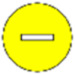	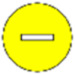	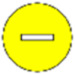	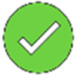	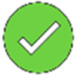
Zhou et al., 2020 [44]	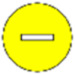	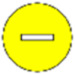	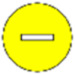	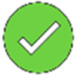	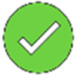
Panda et al., 2016 [45]	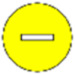	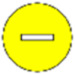	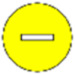	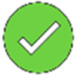	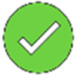
Bassi et al., 2015 [46]	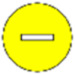	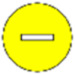	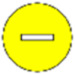	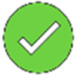	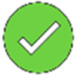
Poli et al., 2020 [47]	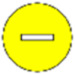	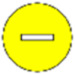	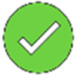	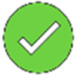	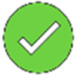
Bhide et al., 2022 [48]	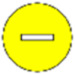	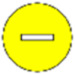	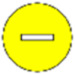	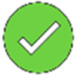	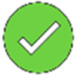
Park et al., 2018 [49]	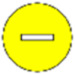	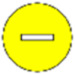	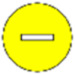	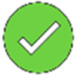	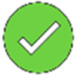
Jensen et al., 2013 [50]	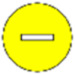	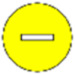	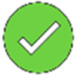	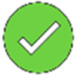	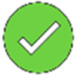
Maeda et al., 2021 [51]	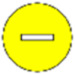	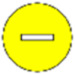	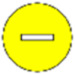	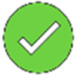	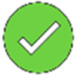
Urban et al., 2021 [52]	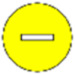	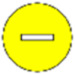	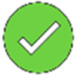	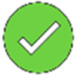	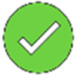
Blume et al., 2021 [53]	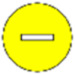	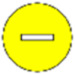	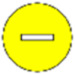	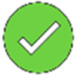	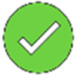
Urban et al., 2022 [54]	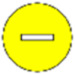	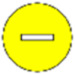	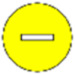	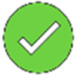	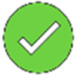
Khojasteh et al., 2019 [55]	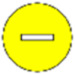	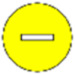	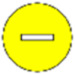	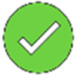	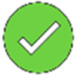
Windisch et al., 2021 [56]	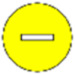	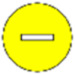	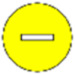	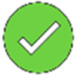	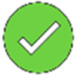

**Table 2:** A summary of the results of studies included in this review.

References (Authors, Year of Publication and Study Details)	Study Methods	Results
Aslan et al., 2020 [9]: 1-year single-blinded randomized controlled clinical trial with 30 periodontal participants, each with one isolated intrabony defect.	Papilla preservation technique with EMD + plus bovine-derived bone substitutes (15 patients—test sites). Papilla preservation technique alone (15 patients—control sites).	Application of papilla preservation technique with and without regenerative biomaterials resulted in significant amounts of CAL gain and PPD reduction, with negligible increase in gingival recession.
Paolantonio et al., 2020 [10]: 1-year triple-blinded randomized controlled clinical trial with 44 periodontal patients exhibiting at least one unfavorable intraosseous defect.	Open flap debridement with PRF associated with ABG (22 patients—test sites). Open flap debridement with EMD + ABG (22 patients—control sites).	PRF-ABG combined treatment of non-contained IBDs produces non-inferior results in terms of CAL gain, PPD reduction, GR increase, and defect bone level gain in comparison with the EMD-ABG combination.
Gautam et al., 2022 [11]: 9-month randomized controlled clinical trial with 39 chronic periodontitis participants, each with one isolated intrabony defect.	Open flap debridement + placebo (13 participants—group A). Open flap debridement + BG + PRF (13 participants—group B). Open flap debridement + ABG + PRF + 1.2 mg Rosuvastatin (13 participants—group C).	Addition of 1.2 mg Rosuvastatin gel, PRF, and ABG has synergistic effects, explaining their role as a regenerative material in the treatment of intrabony defects.
Górski et al., 2020 [12]: 4-year double-blinded randomized controlled clinical split-mouth trial with 15 aggressive periodontitis patients, each with two deep intrabony defects (1 drop-out and 1 tooth extracted due to root fracture).	Papilla preservation technique with xenogenic graft plus MPMs (14 defects—test sites). Papilla preservation technique with xenogenic graft plus standard CMs (13 defects—control sites).	GTR of intrabony defects in aggressive periodontitis with either standard or MPMs yielded similarly successful and maintainable clinical benefits for compromised teeth 4 years following the surgery. The use of MPMs showed no additional benefit.
Gamal et al., 2014 [13]: 1-month single-blinded randomized controlled clinical split-mouth trial with 15 severe chronic periodontitis participants, each with two interproximal contralateral defects.	Open flap debridement with MPMs (15 defects—test sites). Open flap debridement with occlusive membrane (15 defects—control sites).	MPMs coverage of periodontal defects is associated with a significant initial increase in GCF levels of BMP-2, a factor that could improve the clinical outcomes of guided tissue regenerative surgery.
Aggour et al., 2017 [14]: 6-month single-blinded randomized controlled clinical split-mouth trial with 16 generalized aggressive periodontitis patients, each with paired contralateral intrabony defects.	Open flap debridement with ABG mixed with xenograft + PRF (16 defects—test sites). Open flap debridement with composite bone graft + CMs (16 defects—control sites).	PRF has shown favorable results that are comparable to CMs for treatment of intrabony periodontal defects in patients with generalized aggressive periodontitis. Better results concerning gingival margin have been reported when PRF was used.
Hazari et al., 2021 [15]: 6-month randomized controlled clinical trial with 20 chronic generalized periodontitis participants, each with intrabony defects.	Open flap debridement with premixed composite of bioactive calcium phosphosilicate particles and an absorbable synthetic binder (group A). Open flap debridement with premixed composite of bioactive calcium phosphosilicate particles and an absorbable synthetic binder along with PRF (group B).	Evaluation of efficacy of premixed composite of bioactive calcium phosphosilicate particles and an absorbable synthetic binder along with PRF produced more favorable results in relative attachment level gain and more reduction in probing pocket depth when compared to premixed composite alone.
Cieplik et al., 2018 [16]: 13-year double-blinded randomized controlled clinical split-mouth trial with 22 periodontal patients, each with two deep contralateral intrabony defect.	Open flap debridement with β-TCP and bio-resorbable membranes with the additional application of APC (11 defects). Open flap debridement with β-TCP and patient blood (11 defects).	CAL gain following GTR can be maintained over 13 years. The additional use of APC had no positive influence on the long-term stability.
Temraz et al., 2019 [17]: 6-month triple-blinded randomized controlled clinical trial with 22 severe chronic periodontitis participants, each with one intrabony defect.	Open flap debridement and amnion chorion membrane (11 defects—test sites). Open flap debridement and demineralized bone matrix putty (11 defects—control sites).	Amnion chorion membrane barrier and demineralized bone matrix putty allograft provided significant improvement in clinical and radiographic outcomes after 6 months, yet no significant differences were noticed between them.
Ferrarotti et al., 2018 [18]: 1-year double-blinded randomized controlled clinical trial with 29 chronic periodontitis patients presenting one deep intrabony defect.	Minimally invasive surgical technique using micrografts rich in autologous dental pulp stem cells seeded onto collagen sponge (15 defects—test sites). Minimally invasive surgical technique using collagen sponge alone (14 defects—control sites).	Application of dental pulp stem cells significantly improved clinical parameters of periodontal regeneration 1 year after treatment.
Chen et al., 2016 [19]: 1-year single-blinded randomized controlled clinical trial with 30 periodontal participants and a total of 48 intrabony defects.	GTR and autologous periodontal ligament stem cells sheets in combination with xenograft bone substitute (24 defects—test sites). GTR and xenograft bone substitute (24 defects—control sites).	Autologous periodontal ligament stem cells to treat periodontal intrabony defects is safe and does not produce significant adverse effects.
Gonçalves et al., 2008 [20]: 3-week randomized controlled clinical trial with 22 intrabony defects in 22 periodontal patients.	Scaling and root planing with the removal of granulation tissue and root cementum and soft microbial deposits by cleaning the root surface with a microbrush and saline solution (11 defects—test sites). Scaling and root planing with the removal of granulation tissue and root cementum (11 defects—control sites).	mRNA levels for platelet-derived growth factor-alpha, bone sialoprotein, and basic fibroblast growth factor were higher in the sites where root cementum was kept in place compared to the sites where root cementum was removed completely as part of the periodontal therapy. Root cementum may modulate the expression of growth and mineral-associated factors during periodontal regeneration.
Rani et al., 2018 [21]: 6-month randomized controlled clinical trial with 20 mandibular grade II furcation defects in 20 participants.	Open flap debridement with β-TCP with PRF membrane (10 defects—test sites). Open flap debridement with β-TCP alone (10 defects—control sites).	The combination of PRF with β-TCP allograft led to more favorable improvement in the management of grade II furcation defect except PPD.
Bajaj et al., 2013 [22]: 9-month double-blinded randomized controlled clinical trial with 72 mandibular grade II furcation defects in 72 patients.	Open flap debridement with PRF (24 defects—test sites). Open flap debridement with autologous PRP (25 defects—test sites). Open flap debridement alone (23 defects—control sites).	The use of autologous PRF or PRP were both effective in the treatment of furcation defects with uneventful healing of sites.
Queiroz et al., 2016 [23]: 1-year single-blinded randomized controlled clinical trial with 41 mandibular grade II furcation defects in 41 participants.	Open flap debridement with β-TCP + hydroxyapatite (14 defects—test sites). Open flap debridement with EMD + β-TCP + hydroxyapatite (14 defects—test sites). EMD (13 defects—control sites).	EMD + β-TCP + hydroxyapatite does not provide a significant advantage when compared to the isolated approaches. All three tested treatments promote significant improvements and partial closure of class II buccal furcation defects. EMD may be considered an attractive option for this type of defect
Pajnigara et al., 2017 [24]: 6-month double-blinded randomized controlled clinical trial with 20 grade II furcation defects in 20 participants.	Open flap debridement with DFDBA + amnion membrane (10 defects—test sites). Open flap debridement with DFDBA (10 defects—control sites).	DFDBA used with amnion membrane resulted in significant improvement in clinical and radiographic parameters when compared with DFDBA alone.
Huidrom et al., 2022 [25]: 6-month single-blinded randomized controlled clinical trial with 20 mandibular grade II furcation defects in 20 patients.	Open flap debridement with concentrated GF mixed with inorganic bovine bone along with GTR (10 defects—test sites). Open flap debridement with inorganic bovine bone along with GTR (10 defects—control sites).	The use of CGF showed a positive additive efficacy in enhancing the events of periodontal regeneration in the treatment of Degree II mandibular molar furcation defect.
Sneha et al., 2021 [26]: 6-month single-blinded randomized controlled clinical trial with 32 grade II furcation defects in 32 participants.	Open flap debridement with BMP-2 impregnated with absorbable collagen sponge (16 defects—test sites). Open flap debridement with PRF (16 defects—control sites).	The unique regenerative potential BMP-2 impregnated with absorbable collagen sponge makes it a potential agent to be used as a graft material for the treatment of grade II furcation defects.
Isehed et al., 2018 [27]: 5-year double-blinded randomized controlled clinical trial with 25 peri-implantitis patients.	Open flap debridement with EMD (13 patients—test group). Open flap debridement alone (12 patients—control group).	Adjunctive EMD is positively associated with implant survival up to 5 years.
Lee et al., 2020 [28]: 5-month single-blinded randomized controlled clinical trial with 28 post-extraction alveoli in 28 participants following posterior maxillary alveolar ridge preservation.	Extraction sockets filled with DBBM and CM with EMD (10 patients—test group). Extraction sockets filled with DBBM and CM without EMD (10 patients—test group). Spontaneous healing extraction sockets (8 patients—control group).	EMD failed to provide additional benefits in posterior maxillary alveolar ridge preservation in the posterior maxilla.
Jo et al., 2019 [29]: 12-week single-blinded randomized controlled clinical trial with 64 post-extraction alveoli in 64 patients following maxillary alveolar ridge preservation.	Extraction sockets filled with BMP-2-soaked absorbable collagen sponge (32 patients—test group). Extraction sockets filled with β-TCP and hydroxyapatite particles (32 patients—control group).	The delivery systems showed similar efficacy for alveolar ridge preservation without severe adverse events.
Stumbras et al., 2021 [30]: 3-month double-blinded randomized controlled clinical trial with 40 post-extraction alveoli in 40 participants following anterior maxillary alveolar ridge preservation.	Extraction sockets filled with DBBM covered with resorbable native CM (10 patients—test group). Extraction sockets filled with DFDBA covered with resorbable native CM (10 patients—test group). Extraction sockets filled with FGF-2 alone (10 patients—test group). Spontaneous healing extraction sockets (10 patients—control group).	Alveolar ridge preservation technique in the esthetic zone using natural DBBM covered with resorbable native CM or using FGF-2 beneficial to reduce horizontal and vertical bone changes.
Saito et al., 2021 [31]: 16-week single-blinded randomized controlled clinical trial with 45 post-extraction alveoli in 45 patients following alveolar ridge preservation.	Extraction sockets filled with alloplastic graft PLGA coated β-TCP (24 patients—test group). Extraction sockets filled with DFDBA particles covered with a rapidly absorbable collagen dressing (21 patients—control group).	Although a higher proportion of mineralized tissue was associated with the use of DFDBA particles covered with a rapidly absorbable collagen dressing compared to alloplastic graft PLGA-coated β-TCP, both approaches rendered comparable outcomes in terms of maintenance of alveolar bone dimensions.
Gonshor et al., 2011 [32]: 9-month randomized controlled clinical trial with 21 sinus augmentation in 18 participants.	Sinus-augmentation procedures using allograft cellular bone matrix (13 sinus augmentation procedures—test sites). Sinus-augmentation procedures using conventional allograft (8 sinus augmentation procedures—control sites).	The high percentage of vital bone content, after a relatively short healing phase, may encourage a more rapid initiation of implant placement or restoration when a cellular grafting approach is considered.
Majzoub et al., 2020 [33]: retrospective cohort study, with a 1–9.6-year follow-up on 83 patients with 98 treated grade III furcation defects.	GTR using an allogeneic cancellous bone graft and covered by an absorbable membrane.	GTR using allogeneic cancellous bone graft and absorbable collagen membrane to be a viable option for treating furcation-involved teeth if the defect morphology and the location of the defect are favorable.
Canullo et al., 2019 [34]: prospective clinical trial, with a 12-month follow-up on 6 participants with 6 circumferential and semi-circumferential defects due to peri-implantitis.	GBR technique using 50:50 mixture of ABG and allograft and CMs.	The proposed technique might represent a promising result for treatment of circumferential and semi-circumferential bone defects around implants affected by peri-implantitis.
Kadkhodazadeh et al., 2021 [35]: retrospective pilot study, with a 8-month follow-up on 7 patients with 11 peri-implant defects.	GBR using titanium mesh, a combination of ABG, allogenic graft material, and acellular dermal matrix. Soft tissue augmentation and vestibuloplasty were performed in the second-stage surgery, if required.	This technique may lead to promising outcomes in cautiously selected patients seeking to retain their failing implants.
Chiapasco et al., 2020 [36]: retrospective longitudinal cohort study, with a 3–16-year follow-up on 75 participants with 82 grafted bone defects and 182 implants placed post-regeneration bone.	Reconstructive bone procedure with autogenous mandibular bone blocks and rehabilitated with implant-supported prostheses.	Implants placed in areas reconstructed with mandibular bone blocks presented survival rates consistent with those obtained for implants placed in native bone.
Beretta et al., 2021 [37]: clinical and radiological prospective study, with a 5-year follow-up on 8 post-extraction alveoli in 8 patients.	Alveolar socket preservation with DBBM particles covered with a porcine-derived non-cross-linked collagen matrix.	Alveolar socket preservation using DBBM in combination with a porcine-derived non-cross-linked collagen matrix proved to be an effective technique to maintain stable dimensional volumes of both hard and soft tissues.
Manavella et al., 2018 [38]: retrospective cohort study, with a 12-month follow-up on 11 severely resorbed alveolar sockets in 11 participants.	Ridge augmentation procedure with DBBM with 10% collagen covered with a CMs.	A ridge preservation technique performed with DBBM, and a CMs was able to improve ridge shape and dimensions in compromised alveolar sockets.
Zafiropoulos et al., 2020 [39]: retrospective clinical study, with a 6-month follow-up on 44 post-extraction premolar alveoli in 44 patients.	Alveolar socket preservation with non-resorbable dense polytetrafluoroethylene membranes.	The use of the examined new non-resorbable dense polytetrafluoroethylene membranes consistently led to the preservation of hard tissue in the extraction sites.
Beretta et al., 2015 [40]: retrospective long-term follow-up study, with a 78-month follow-up on 61 participants with 192 implants placed after guided bone regeneration.	Assessing the CSR after GBR using DBBM (76 implants). Assessing the CSR after GBR using ABG (20 implants). Assessing the CSR after GBR using 1:1 ratio mixture of ABG and demineralized DBBM (96 implants). Assessing the CSR after GBR using resorbable membranes (101 grafted sites). Assessing the CSR after GBR using non-resorbable membranes (91 grafted sites).	All the procedures performed with different bone grafts and type of membranes guaranteed optimal results in CSR (> 90%).
Yoshikawa et al., 2020 [41]: case report, with an 18-month follow-up on a 57-year-old man participant with generalized chronic periodontitis.	Surgical periodontal therapy with using EMD and ABG.	Periodontal regenerative therapy using EMD with ABG resulted in improvement in vertical bone resorption.
Thakkalapati et al., 2015 [42]: case report, with a 2-year follow-up on a 30-year-old female patient with one-wall intrabony osseous defect.	Open flap debridement and placement of combination of autologous PRP and DBBM.	Radiographic evidence of bone formation was observed as early as 3 months with almost complete fill by 6 months post-operatively. The results were maintained over a period of 2 years.
Pal et al., 2018 [43]: case report, with a 1-year follow-up on a male participant with a mandibular torus and intrabony defect at the mandibular right central incisor.	ABG was obtained from a mandibular torus and utilized to fill the IBD.	The mandibular torus provided sufficient graft material and eliminated the need for a second surgical site. A follow-up at 1 year revealed reduction in clinical attachment loss and complete resolution of tooth mobility.
Zhou et al., 2020 [44]: case report, with a 12-month follow-up on a 56-year-old and 34-year-old female patients with mandibular grade III furcation involvement.	Open flap debridement with allogenic bone graft and PRF.	Successful periodontal regeneration of grade III furcation defects can be achieved by using PRF in combination with bone allograft.
Panda et al., 2016 [45]: case report, with a 6-month follow-up on a 36-year-old male participant with class III gingival recession defect along with furcation involvement.	Open flap debridement with PBFP.	PBFP may be considered as a reliable modality for root coverage of severe GR defects, which could not be repaired by other conventional procedures.
Bassi et al., 2015 [46]: case report, with a 17-year follow-up on a 48-year-old female patient with peri-implantitis affecting the mandibular right second molar.	Open flap debridement with DBBM and a CM.	The treatment seemed to show improved clinical results up to a relevant follow-up period.
Poli et al., 2020 [47]: case series, with a 9-month follow-up with reentry on 6 participants with peri-implantitis affecting maxillary or mandibular area.	Open flap debridement, antimicrobial photodynamic therapy with a solution of phenothiazine chloride dye consisting of methylenthioniniumchlorid photo-activated with 100-mW diode Laser (660 ± 10 nm) and regeneration with 70:30 mixture of ABG and DBBM particles, titanium mesh, and resorbable CM.	At the reentry surgery, no evidence of granulation tissue, purulence, or progressive bone resorption was found. Newly formed bonelike tissue hardly distinguishable from the existing bone was found in intimate contact with the implant surface at most of the implant sites. A modest amount of augmented bone was obtained at only one mandibular site, resulting in persistent dehiscence-type defects associated with a supracrestal component.
Bhide et al., 2022 [48]: case report, with a 2-year follow-up on a 68-year-old male participant with peri-implantitis affecting the endosseous implant that replaced the maxillary left first molar.	GBR performed using DBBM covered with a dehydrated human deepithelialized human amnion-chorion membrane.	Posttreatment clinical assessment suggested that the patient responded well to surgical regenerative therapy, with the reestablishment of healthy peri-implant soft tissues. It is possible to treat peri-implantitis successfully and obtain stable long-term results with a GBR approach utilizing a DBBM covered with a dehydrated human deepithelialized human amnion-chorion membrane.
Park et al., 2018 [49]: case report, with a 27-month follow-up on a 52-year-old male patient with peri-implantitis affecting mandibular right second premolar area.	Open flap debridement with bovine-derived hydroxyapatite and EMD.	The area showed maintenance of graft material with increased radiopacity around the dental implant.
Jensen et al., 2013 [50]: case series, with a 6-month follow-up on 4 participants with posterior maxillary peri-implantitis/sinusitis and occult peri-implant oroantral fistulae.	Sinus floor grafting with BMP-2 in an absorbable collagen sponge carrier.	Sinus floor grafting to salvage trans-sinus implants or to prevent peri-implantitis/sinusitis is suggested by the successful use of BMP-2 in an absorbable collagen sponge carrier.
Maeda et al., 2021 [51]: case series, with a 6-month follow-up on 16 patients with 16 post-extraction alveoli.	Alveolar ridge preservation a DBBM and anodized titanium foil.	The application of a DBBM covered with anodized titanium foil resulted in clinically important horizontal preservation of the alveolar ridge at 6 months after extraction.
Urban et al., 2021 [52]: case series, with a 10-year follow-up on 86 participants with 92 sinus lifts and 209 implants placed.	Sinus augmentation using particulate ABG and inorganic bovine bone-derived mineral (3:7 ratio). Implants were placed on baseline residual alveolar ridge height.	Implant placement after two-stage sinus grafting utilizing the sagittal sandwich technique is a relatively safe and predictable procedure with minimal complications and marginal bone loss after 10-year follow-up.
Blume et al., 2021 [53]: case report, with a 6-month follow-up on a 19-year-old male patient with a complex maxillary defect.	Reconstruction of a complex maxillary defect using allogeneic bone block.	The present case report demonstrates a limited resorption of the allogeneic bone block and further emphasizes the practicability of determining bone resorption by the here introduced method.
Urban et al., 2022 [54]: case report, with a 3-month follow-up on a 55-year-old male participant with peri-implantitis around an implant in the maxillary left central incisor position and with severe bone loss on the mesial aspect of the maxillary left lateral incisor.	Ridge augmentation using composite bone graft (ABG + xenograft particles), and a PDGF. Tissue augmentation with connective tissue grafts. Peri-implant keratinez mucosa augmentation using labial gingival graft strip and a xenogenic collagen matrix.	Simultaneous vertical ridge augmentation and periodontal regeneration can be achieved to manage a challenging clinical situation.
Khojasteh et al., 2019 [55]: case report, with a 10-month and 6-month follow-up, respectively, on a 19-year-old female and 22-year-old male participants with large alveolar defects.	GBR using adipose-derived stem cells, originated from buccal fat pad, loaded with natural bovine bone mineral.	The application of adipose-derived stem cells isolated from buccal fat pad in combination with natural bovine bone mineral can be considered an efficient treatment for bone regeneration in large alveolar bone defects.
Windisch et al., 2021 [56]: prospective case series, with a 9-month follow-up on 19 patients with 24 vertical alveolar ridge defects.	Vertical GBR consisting of a split-thickness flap and using titanium-reinforced high-density polytetrafluoroethylene membrane combined with particulated ABG and bovine-derived xenograft in 1:1 ratio.	Vertical GBR consisting of a split-thickness flap and using titanium-reinforced non-resorbable membrane in conjunction with a 1:1 mixture of particulated ABG and bovine-derived xenograft may lead to a predictable vertical and horizontal hard tissue reconstruction.

**Table 3:** Summary of the most relevant growth factors and their origin, composition, mechanism of action, and indications. Adapted from Ref. [82]. Copyright © 2015 Fernando Suarez-Lopez del Amo et al.

Agent	rhPDGF-BB	EMD	PRP/PRF	FGF-2	BMP-2
**Origin**	Blood platelets	Hertwig’s epithelial root sheath	Platelet alpha granules	Fibroblast growth factors family	Recombinant DNA biotechnology using mammalian cells
**Composition**	Protein	90% Amelogenin	PDGF, I-LGF, VEGF, TGF-beta	Protein	Bone Morphogenic Protein-2
**Mechanism** **of action**	Chemotaxis and mitogenesis	Precise mechanism of action still unknown	Combination of different mechanism of different growth factors contained with the platelet concentrates (e.g., revascularization, fibrogenesis, etc.)	Proliferation PDL cells Migration PDL cells Differentiation PDL cells ECM production	Increase of proliferation, mineralization, and expression of alkaline phosphatase and osteocalcin
**Clinical indications**	Intrabony defects; Furcations; Gingival recession defects	Intrabony defects; Optimize tissue height in esthetic zone	Recession coverage Barrier membrane	Peri-implant defects Intrabony defects	Use in case of systemic or anatomic or conditions where successful bone regeneration cannot be achieved with conventional grafts

Legend: rhPDGF-BB: Recombinant Human Platelet-Derived Growth Factor-BB; EMD: Enamel Matrix Derivate; PRP/PRF: Platelet-Rich Plasma/Platelet-Rich Fibrin; FGF-2: Fibroblast Growth Factor-2; BMP-2: Bone Morphogenic Protein-2.

## Data Availability

Upon request to the corresponding author, the data are available for use.

## Supplementary Materials

The following supporting information can be downloaded at: https://www.mdpi.com/article/10.3390/bioengineering9100594/s1, Table S1: Summary table of studies excluded in this review.; Table S2: Criteria for judging risk of bias in the “Risk of bias” assessment tool.; Table S3: NHLBI Quality Assessment of Controlled Intervention Studies.; Table S4: NHLBI Quality Assessment Tool for Observational Cohort and Cross-Sectional Studies; Table S5: NHLBI Quality Assessment Tool for Case Series Studies/Case Reports.

## Protocol Registration

No protocol registration has been conducted.

## Abbreviations

ABG	autogenous bone graft
APC	autogenous platelet concentrates
BMP-2	bone morphogenetic protein-2
BMP	bone morphogenetic proteins
BoP	bleeding on probing
CAL	clinical attachment level
CMs	collagen membranes
CSR	cumulative survival rate
DBBM	demineralized bovine bone mineral grafts
DFDBA	freeze-dried demineralized bone allograft
EMD	enamel matrix derivative
FGF-2	fibroblast growth factor-2
GBR	guided bone regeneration
GF	growth factors
GR	gingival recession
GTR	guided tissue regeneration
IBDs	intrabony defects
MeSH	medical subject heading
MPMs	modified perforated membranes
NHLBI	national heart, lung, and blood Institute
PBFP	pedicled buccal fat pad
PDGF	platelet-derived growth factor
PI	plaque index
PLGA	poly-lactic-glycolic acid
PPD	probing pocket depth
PRF	platelet-rich fibrin
PRP	platelet-rich plasma
β-TCP	β-tricalcium phosphate allograft
