# Supplementary material for: Latest Findings of the Regenerative Materials Application in Periodontal and Peri-Implant Surgery: A Scoping Review
Source: Bioengineering (Basel). 2022 Oct 21;9(10):594. doi: 10.3390/bioengineering9100594 (PMC9598513; doi:10.3390/bioengineering9100594)
Supplement: Supplementary file 1 [file bioengineering-09-00594-s001.zip › bioengineering-1931746-supplementary.pdf]

Table S1. Summary table of studies excluded in this review.

Excluded Studies	Exclusion Reasons
Wu et al., 2021 [57]	Narrative Review
Correia et al., 2018 [58]	Systematic Review
Avila-Ortiz et al., 2016 [59]	Systematic Review
Deb et al., 2015 [60]	Narrative Review
Egusa et al., 2012 [61]	Narrative Review
Sancho et al., 2019 [62]	Systematic Review
DeCarlo et al., 2006 [63]	Narrative Review
Tavelli et al., 2021 [64]	Systematic Review
Farimani et al., 2021 [65]	Systematic Review and Meta-Analysis
Ripamonti et al. 2009 [66]	Narrative Review
Goker et al., 2019 [67]	Narrative Review
Marei et al., 2018 [68]	Narrative Review
Fayzullin et al., 2021 [69]	Narrative Review
Liu et al., 2019 [70]	Narrative Review
Xu et al., 2019 [71]	Narrative Review

Rios et al., 2011 [72]	Narrative Review
Iwata et al., 2014 [73]	Narrative Review
Chen et al., 2010 [74]	Narrative Review
Kim et al., 2020 [75]	Narrative Review
Iviglia et al., 2019 [76]	Narrative Review
Carter et al., 2017 [77]	Narrative Review
Kim et al., 2014 [78]	Narrative Review
Khoshkam et al. 2015 [79]	Systematic Review and Meta-Analysis
Jepsen et al., 2020 [80]	Systematic Review and Meta-Analysis
Miron et al., 2017 [81]	Systematic Review

Table S2. Criteria for judging risk of bias in the "Risk of bias" assessment tool.

**Random Sequence Generation**	
Criteria for a judgement of 'Low risk' of bias.	The investigators describe a random component in the sequence generation process.
Criteria for the judgement of 'High risk' of bias.	The investigators describe a non-random component in the sequence generation process. Usually, the description would involve some systematic, non-random approach.<br>Other non-random approaches happen much less frequently than the systematic approaches mentioned above and tend to be obvious. They usually involve judgement or some method of non-random categorization of participants.
**Allocation Concealment**	
Criteria for a judgement of 'Low risk' of bias.	Participants and investigators enrolling participants could not foresee assignment because one of the following, or an equivalent method, was used to conceal allocation.
Criteria for the judgement of 'High risk' of bias.	Participants or investigators enrolling participants could possibly foresee assignments and thus introduce selection bias.
**Blinding**	
Criteria for a judgement of 'Low risk' of bias.	Any one of the following:<br>- No blinding or incomplete blinding, but the review authors judge that the outcome is not likely to be influenced by lack of blinding;<br>- Blinding of participants and key study personnel ensured, and unlikely that the blinding could have been broken;<br>- No blinding of outcome assessment, but the review authors judge that the outcome measurement is not likely to be influenced by lack of blinding;<br>- Blinding of outcome assessment ensured, and unlikely that the blinding could have been broken.
Criteria for the judgement of 'High risk' of bias.	Any one of the following:<br>- No blinding or incomplete blinding, and the outcome is likely to be influenced by lack of blinding;<br>- Blinding of key study participants and personnel attempted, but likely that the blinding could have been broken, and the outcome is likely to be influenced by lack of blinding;

	- No blinding of outcome assessment, and the outcome measurement is likely to be influenced by lack of blinding;<br>- Blinding of outcome assessment, but likely that the blinding could have been broken, and the outcome measurement is likely to be influenced by lack of blinding.
**Incomplete Outcome Data**	
Criteria for a judgement of 'Low risk' of bias.	Any one of the following:<br>- No missing outcome data;<br>- Reasons for missing outcome data unlikely to be related to true outcome (for survival data, censoring unlikely to be introducing bias);<br>- Missing outcome data balanced in numbers across intervention groups, with similar reasons for missing data across groups;<br>- For dichotomous outcome data, the proportion of missing outcomes compared with observed event risk not enough to have a clinically relevant impact on the intervention effect estimate;<br>- For continuous outcome data, plausible effect size (difference in means or standardized difference in means) among missing outcomes not enough to have a clinically relevant impact on observed effect size;<br>- Missing data have been imputed using appropriate methods.
Criteria for the judgement of 'High risk' of bias.	Any one of the following:<br>- Reason for missing outcome data likely to be related to true outcome, with either imbalance in numbers or reasons for missing data across intervention groups;<br>- For dichotomous outcome data, the proportion of missing outcomes compared with observed event risk enough to induce clinically relevant bias in intervention effect estimate;<br>- For continuous outcome data, plausible effect size (difference in means or standardized difference in means) among missing outcomes enough to induce clinically relevant bias in observed effect size;<br>- 'As-treated' analysis done with substantial departure of the intervention received from that assigned at randomization;<br>- Potentially inappropriate application of simple imputation.
**Selective Reporting**	
Criteria for a judgement of 'Low risk' of bias.	Any one of the following:

	- The study protocol is available and all of the study's pre-specified (primary and secondary) outcomes that are of interest in the review have been reported in the pre-specified way;<br>- The study protocol is not available but it is clear that the published reports include all expected outcomes, including those that were pre-specified (convincing text of this nature may be uncommon).
Criteria for the judgement of 'High risk' of bias.	Any one of the following:<br>- Not all of the study's pre-specified primary outcomes have been reported;<br>- One or more primary outcomes is reported using measurements, analysis methods or subsets of the data (e.g., subscales) that were not pre-specified;<br>- One or more reported primary outcomes were not pre-specified (unless clear justification for their reporting is provided, such as an unexpected adverse effect);<br>- One or more outcomes of interest in the review are reported incompletely so that they cannot be entered in a meta-analysis;<br>- The study report fails to include results for a key outcome that would be expected to have been reported for such a study.

Table S3. NHLBI Quality Assessment of Controlled Intervention Studies.

NHLBI Quality Assessment of Controlled Intervention Studies																
**First Author et al., Year**	**Q1**	**Q2**	**Q3**	**Q4**	**Q5**	**Q6**	**Q7**	**Q8**	**Q9**	**Q10**	**Q11**	**Q12**	**Q13**	**Q14**	**Total Score**	**Quality Rating**
Aslan et al., 2020 [9]	Y	Y	Y	Y	Y	Y	Y	Y	N	Y	Y	NR	NR	Y	11/14 (78.57%)	Good
Paolantonio et al., 2020 [10]	Y	Y	Y	Y	Y	Y	Y	Y	N	Y	Y	NR	Y	Y	11/14 (78.57%)	Good
Gautam et al., 2022 [11]	Y	Y	NR	NR	NR	Y	Y	Y	N	Y	Y	NR	Y	Y	8/14 (57.14%)	Fair
Górski et al., 2020 [12]	Y	Y	Y	Y	Y	Y	Y	Y	N	Y	Y	NR	NR	Y	11/14 (78.57%)	Good
Gamal et al., 2014 [13]	Y	Y	NR	Y	Y	Y	Y	Y	N	Y	Y	N	NR	Y	10/14 (71.42%)	Fair
Aggour et al., 2017 [14]	Y	Y	Y	Y	Y	Y	Y	Y	N	Y	Y	N	NR	Y	11/14 (78.57%)	Good
Hazari et al., 2021 [15]	Y	Y	NR	NR	NR	Y	Y	Y	N	Y	Y	N	Y	Y	9/14 (64.28%)	Fair
Cieplik et al., 2018 [16]	Y	Y	Y	Y	Y	Y	Y	Y	N	Y	Y	N	NR	Y	11/14 (78.57%)	Good
Temraz et al., 2019 [17]	Y	Y	Y	Y	Y	Y	Y	Y	N	Y	Y	N	NR	Y	11/14 (78.57%)	Good
Ferrarotti et al., 2018 [18]	Y	Y	Y	Y	Y	Y	Y	Y	N	Y	Y	N	NR	Y	11/14 (78.57%)	Good
Chen et al., 2016 [19]	Y	Y	NR	Y	Y	Y	Y	Y	N	Y	Y	N	Y	Y	11/14 (78.57%)	Good
Gonçalves et al., 2008 [20]	Y	Y	NR	NR	NR	Y	Y	Y	N	Y	Y	NR	Y	Y	10/14 (71.42%)	Fair
Rani et al., 2018 [21]	Y	Y	NR	NR	NR	Y	Y	Y	N	Y	Y	NR	Y	Y	10/14 (71.42%)	Fair
Bajaj et al., 2013 [22]	Y	Y	Y	Y	Y	Y	Y	Y	N	Y	Y	NR	NR	Y	11/14 (78.57%)	Good
Queiroz et al., 2016	Y	Y	Y	Y	Y	Y	Y	Y	N	Y	Y	NR	NR	Y	11/14	Good

[23]															(78.57%)	
Pajnigara et al., 2017 [24]	Y	Y	NR	Y	Y	Y	Y	Y	N	Y	Y	NR	NR	Y	10/14 (71.42%)	Fair
Huidrom et al., 2022 [25]	Y	Y	Y	Y	Y	Y	Y	Y	N	Y	Y	N	NR	Y	11/14 (78.57%)	Good
Sneha et al., 2021 [26]	Y	Y	NR	Y	Y	Y	Y	Y	N	Y	Y	NR	NR	Y	10/14 (71.42%)	Fair
Isehed et al., 2018 [27]	Y	Y	Y	Y	Y	Y	Y	Y	N	Y	Y	NR	NR	Y	11/14 (78.57%)	Good
Lee et al., 2020 [28]	Y	Y	Y	Y	Y	Y	Y	Y	N	Y	Y	N	NR	Y	11/14 (78.57%)	Good
Jo et al., 2019 [29]	Y	Y	Y	Y	Y	Y	Y	Y	N	Y	Y	NR	NR	Y	11/14 (78.57%)	Good
Stumbras et al., 2021 [30]	Y	Y	Y	Y	Y	Y	Y	Y	N	Y	Y	NR	NR	Y	11/14 (78.57%)	Good
Saito et al., 2021 [31]	Y	Y	Y	Y	Y	Y	Y	Y	N	Y	Y	N	NR	Y	11/14 (78.57%)	Good
Gonshor et al., 2011 [32]	Y	Y	NR	NR	NR	Y	Y	Y	N	Y	Y	NR	Y	Y	10/14 (71.42%)	Fair

Q1: Was the study described as randomized, a randomized trial, a randomized clinical trial, or an RCT?, Q2: Was the method of randomization adequate (i.e., use of randomly generated assignment)?, Q3: Was the treatment allocation concealed (so that assignments could not be predicted)?, Q4: Were study participants and providers blinded to treatment group assignment?, Q5: Were the people assessing the outcomes blinded to the participants' group assignments?, Q6: Were the groups similar at baseline on important characteristics that could affect outcomes (e.g., demographics, risk factors, co-morbid conditions)?, Q7: Was the overall drop-out rate from the study at endpoint 20% or lower of the number allocated to treatment?, Q8: Was the differential drop-out rate (between treatment groups) at endpoint 15 percentage points or lower?, Q9: Was there high adherence to the intervention protocols for each treatment group?, Q10: Were other interventions avoided or similar in the groups (e.g., similar background treatments)?, Q11: Were outcomes assessed using valid and reliable measures, implemented consistently across all study participants?, Q12: Did the authors report that the sample size was sufficiently large to be able to detect a difference in the main outcome between groups with at least 80% power?, Q13: Were outcomes reported or subgroups analyzed prespecified (i.e., identified before analyses were conducted)?, Q14: Were all randomized participants analyzed in the group to which they were originally assigned, i.e., did they use an intention-to-treat analysis?; Total Score: Number of yes; CD: cannot be determined; NA: not applicable; NR: not reported; N: no; Y: yes. Quality Rating: Poor <50%, Fair 50–75%, Good ≥75%.

Table S4. NHLBI Quality Assessment Tool for Observational Cohort and Cross-Sectional Studies.

	NHLBI Quality Assessment Tool for Observational Cohort and Cross-Sectional Studies														
**First Author et al., Year**	**Q1**	**Q2**	**Q3**	**Q4**	**Q5**	**Q6**	**Q7**	**Q8**	**Q9**	**Q10**	**Q11**	**Q12**	**Q13**	**Q14**	**Total Score**	**Quality Rating**
Majzoub et al., 2020 [33]	Y	Y	Y	Y	N	Y	Y	N	Y	Y	Y	N	Y	Y	11/14 (78.57%)	Good
Canullo et al., 2019 [34]	Y	Y	Y	Y	N	Y	Y	N	Y	Y	Y	N	Y	Y	11/14 (78.57%)	Good
Kadkhodazadeh et al., 2021 [35]	Y	Y	Y	Y	N	Y	N	Y	Y	Y	Y	N	Y	Y	10/14 (71.42%)	Fair
Chiapasco et al., 2020 [36]	Y	Y	Y	Y	N	Y	Y	Y	Y	Y	Y	N	Y	Y	12/14 (85.71%)	Good
Beretta et al., 2021 [37]	Y	Y	Y	Y	N	Y	Y	Y	Y	Y	Y	N	Y	Y	12/14 (85.71%)	Good
Manavella et al., 2018 [38]	Y	Y	Y	Y	N	Y	Y	N	Y	Y	Y	N	Y	Y	11/14 (78.57%)	Good
Zafiropoulos et al., 2020 [39]	Y	Y	Y	Y	N	Y	N	N	Y	Y	Y	N	Y	Y	10/14 (71.42%)	Fair
Beretta et al., 2015 [40]	Y	Y	Y	Y	N	Y	Y	N	Y	Y	Y	N	Y	Y	11/14 (78.57%)	Good

Q1: Was the research question or objective in this paper clearly stated?, Q2: Was the study population clearly specified and defined?, Q3: Was the participation rate of eligible persons at least 50%?, Q4: Were all the subjects selected or recruited from the same or similar populations (including the same time period)? Were inclusion and exclusion criteria for being in the study prespecified and applied uniformly to all participants?, Q5: Was a sample size justification, power description, or variance and effect estimates provided?, Q6: For the analyses in this paper, were the exposure(s) of interest measured prior to the outcome(s) being measured?, Q7: Was the timeframe sufficient so that one could reasonably expect to see an association between exposure and outcome if it existed?, Q8: For exposures that can vary in amount or level, did the study examine different levels of the exposure as related to the outcome (e.g., categories of exposure, or exposure measured as continuous variable)?, Q9: Were the exposure measures (independent variables) clearly defined, valid, reliable, and implemented consistently across all study participants?, Q10: Was the exposure(s) assessed more than once over time?, Q11: Were the outcome measures (dependent variables) clearly defined, valid, reliable, and implemented consistently across all study participants?, Q12: Were the outcome assessors blinded to the exposure status of participants?, Q13: Was loss to follow-up after baseline 20% or less?, Q14: Were key potential confounding variables measured and adjusted statistically for their impact on the relationship between exposure(s) and outcome(s)?; Total Score: Number of yes; CD: cannot be determined; NA: not applicable; NR: not reported; N: no; Y: yes. Quality Rating: Poor <50%, Fair 50 – 75%, Good ⩾75%.

Table S5. NHLBI Quality Assessment Tool for Case Series Studies/Case Reports.

NHLBI Quality Assessment Tool for Case Series/Case Reports Studies											
**First Author et al., Year**	**Q1**	**Q2**	**Q3**	**Q4**	**Q5**	**Q6**	**Q7**	**Q8**	**Q9**	**Total Score**	**Quality Rating**
Yoshikawa et al., 2020 [41]	Y	Y	N	N	Y	Y	Y	N	Y	6/9 (66.67%)	Fair
Thakkalapati et al., 2015 [42]	Y	Y	N	N	Y	Y	Y	N	Y	6/9 (66.67%)	Fair
Pal et al., 2018 [43]	Y	Y	N	N	Y	Y	Y	N	Y	6/9 (66.67%)	Fair
Zhou et al., 2020 [44]	Y	Y	N	N	Y	Y	Y	N	Y	6/9 (66.67%)	Fair
Panda et al., 2016 [45]	Y	Y	N	N	Y	Y	N	N	Y	5/9 (55.55%)	Fair
Bassi et al., 2015 [46]	Y	Y	N	N	Y	Y	Y	N	Y	6/9 (66.67%)	Fair
Poli et al., 2020 [47]	Y	Y	Y	Y	Y	Y	N	Y	Y	8/9 (88.89%)	Good
Bhide et al., 2022 [48]	Y	Y	N	N	Y	Y	Y	N	Y	6/9 (66.67%)	Fair
Park et al., 2018 [49]	Y	Y	N	N	Y	Y	Y	N	Y	6/9 (66.67%)	Fair
Jensen et al., 2013 [50]	Y	Y	Y	Y	Y	Y	N	Y	Y	8/9 (88.89%)	Good
Maeda et al., 2021 [51]	Y	Y	N	N	Y	Y	N	N	Y	5/9 (55.55%)	Fair
Urban et al., 2021 [52]	Y	Y	Y	Y	Y	Y	Y	Y	Y	9/9 (100%)	Good
Blume et al., 2021 [53]	Y	Y	N	N	Y	Y	N	N	Y	5/9 (55.55%)	Fair
Urban et al., 2022 [54]	Y	Y	N	N	Y	Y	N	N	Y	5/9 (55.55%)	Fair

Khojasteh et al., 2019 [55]	Y	Y	N	N	Y	Y	N	N	Y	5/9 (55.55%)	Fair
Windisch et al., 2021 [56]	Y	Y	Y	Y	Y	Y	N	Y	Y	8/9 (88.89%)	Good

Q1: Was the study question or objective clearly stated?, Q2: Was the study population clearly and fully described, including a case definition?, Q3: Were the cases consecutive?, Q4: Were the subjects comparable?, Q5: Was the intervention clearly described?, Q6: Were the outcome measures clearly defined, valid, reliable, and implemented consistently across all study participants?, Q7: Was the length of follow-up adequate?, Q8: Were the statistical methods well-described?, Q9: Were the results well-described?; Total Score: Number of yes; CD: cannot be determined; NA: not applicable; NR: not reported; N: no; Y: yes. Quality Rating: Poor <50%, Fair 50–75%, Good ≥75%.

Search strategy on PubMed (MEDLINE) and Scopus

#1 “Alveolar Ridge Augmentation” [MESH] OR (Alveolar Ridge Augmentations) OR (Mandibular Ridge Augmentation) OR (Maxillary Ridge Augmentation)

#2 “Regenerative Medicine” [MESH] OR (Regenerative Medicines) OR (Biocompatible Materials) OR (Tissue Engineering)

#3 “Furcation defects” [MESH] OR (Furcation defect)

#4 “Peri-Implantitis” [MESH] OR (Periimplantitis)

#5 “Alveolar bone loss” [MESH] OR (Alveolar Bone Losses) OR (Alveolar Process Atrophy) OR (Alveolar Resorption) OR (Periodontal Bone Loss) OR (Periodontal Resorption) OR (Alveolar Bone Atrophy)

#6 #1 AND #2

#7 #3 AND #2

#8 #4 AND #2

#9 #5 AND #2
